# Developing a responsive model to societal needs in medical education

**DOI:** 10.1186/s12909-024-05355-9

**Published:** 2024-04-04

**Authors:** Hooman Khanpoor, Mohammad Amerzadeh, Ahad Alizadeh, Omid Khosravizadeh, Sima Rafiei

**Affiliations:** 1 Student Research Committee, School of Health, Qazvin University of Medical Sciences, Qazvin, Iran; 2 Social Determinants of Health Research Center, Research Institute for Prevention of Non- Communicable Diseases, Qazvin University of Medical Sciences, Qazvin, Iran

**Keywords:** Responsiveness, Health system, Iran, Education

## Abstract

**Background:**

Responsiveness is relevant in the context of treatment and the provision of medical services. However, if we delve deeper into the subject, we must establish and develop responsiveness within the medical sciences education system. This study aims to identify the dimensions that significantly impact responsiveness in the medical education system based on a comprehensive review and expert opinions in healthcare.

**Methods:**

The present research is descriptive-analytical in terms of its objective and follows a mixed-method approach. This study was conducted in three stages. Initially, we utilized relevant keywords related to education in databases, such as Web of Science, Scopus, ScienceDirect, OVID, CINHAL, EBSCO, Google Scholar, Iranmedex, SID, and Irandoc. Subsequently, in the expert panel session stage, the factors influencing responsiveness were identified in the comprehensive review stage, and with this thematic background, they were conceptualized. Finally, the Confirmatory Factor Analysis (CFA) technique was employed to coherently examine the relationships between variables and present the final model.

**Results:**

We obtained 32 articles from the comprehensive review of studies. Four components in planning, implementation, monitoring and evaluation, and intersectoral cooperation were identified based on expert panel opinions. Based on the standardized coefficients, the components of research-based educational planning, community-oriented education evaluation indicators, and utilization of modern educational methods are statistically significant.

**Conclusion:**

The establishment and development of responsiveness in the medical sciences education system involve training specialized and responsive human resources through innovative educational methods that have sufficient familiarity with the multidimensional concepts of health and how to achieve them. This approach allows for practical and responsible steps toward training competent and committed physicians in line with the needs of society. On the other hand, responsiveness in the medical sciences education system can be improved by enhancing research-based educational planning and developing community-oriented evaluation indicators that can assess the number of revised educational programs based on societal needs. Therefore, understanding the critical elements in revising medical education programs, which play the most significant role in addressing societal needs and responding to changing disease patterns and new health priorities, is both a necessity and an important priority.

## Background

Given rapid changes in the healthcare needs of societies and the need to improve population health, there is an increasing need to enhance the responsiveness of healthcare systems as a critical element in achieving health equity and meeting individuals’ health needs [1]. Many European countries have recognized that the future of healthcare systems relies on their ability to respond more effectively to society’s rapidly changing needs and make informed decisions to improve their multi-dimensional needs [2]. Responsiveness is not confined to the context of treatment and the provision of healthcare services; instead, it expands its applicability to other service domains, including health, medical education, and related research [3]. According to the definition given by the World Health Organization (WHO), medical schools have an essential responsibility to direct their education, research, and service provision in line with the community health priorities [4]. Active engagement in responding to the population’s social needs is a critical social accountability that medical schools must follow [4]. There are different priorities for social responsiveness in the medical education system that mainly concentrate on teaching a necessary skill set to trainees in order to enable them to meet population health, integrate Social Determinants of Health (SDH) in educational planning, and address health inequalities in the society [5, 6]. Furthermore, ensuring a supportive learning and working environment that enables the provision of adequate teaching while providing safe care to service recipients in settings where trainees can learn and provide healthcare services simultaneously is another critical priority [7]. Social accountability is essential for all human beings to enjoy the four values of justice in education, educational quality, effective interactions between the provision of healthcare services and the field of education, and finally, creating maximum efficiency in the provision of health and treatment services, which can bring many beneficial effects for the society and their wellbeing [8].

Literature affirms that the curriculum of many study fields should move towards responsive education to provide a suitable condition for increasing the compatibility between theory and practice [9]. Studies also highlighted that adding courses on SDH to the medical curriculum can improve students’ knowledge and awareness regarding the relevant concepts and consequently provide more comprehensive services [10]. A literature review shows that the Iranian education system lacks determined strategies for developing trainees based on society’s needs and SDH. In addition, the system is facing the challenge of educating capable graduates who should respond to the health needs and current challenges of society in an effective manner. In some cases, there is no mechanism to improve their knowledge, awareness, and motivation to focus on the needs assessment of the community [8]. Thus, integrating medical education into the provision of healthcare services was considered one of the most practical strategies for the responsiveness of healthcare systems [11]. Accordingly, the medical curriculum needs to be revised to provide beneficial opportunities for trainees to learn the required skills to provide high-quality care and support.To this end, the recognition of essential elements in the revision of medical education programs in such a way that it plays the most significant role in addressing societal needs and responding to changing disease patterns and new health priorities has become both a necessity and a crucial priority [11, 12].

In other words, to establish a responsive medical education system, especially from the social aspect, institutions must engage in the training of specialized and skilled human resources who can actively cooperate with communities, the government, health systems, and social entities to deal with healthcare inequalities in an effective way [12]. The primary mission of social accountability is to obtain the maximum possible points for the accreditation of educational organizations, acquire necessary licenses and certificates, establish competency-based training systems and continuous professional development, comply with the principles of differentiation of expertise in medical education, raise standards in teaching and learning, move towards more creativity in educational methods and respond to societal needs more effectively. Through these strategies, the goal of educating graduates who are aware of the social needs of society can be realized [13]. In addition, social accountability criteria are still being formed and tested to enable medical schools to guide education in a targeted way and in relation to the social determinants of health [14]. Our study aims to identify a comprehensive set of features that significantly impact the responsiveness of the medical education system based on a mixed-method study. The study also focuses on identifying and conceptualizing these dimensions within the framework of the final model.

## Methods

### Study design and participants

This mixed-method study uses quantitative and qualitative methods to develop a responsive model in medical education. A literature review was conducted in the initial phase of the study to gain a comprehensive understanding of different domains regarding medical education. Afterward, researchers set up an expert panel to finalize the domains from the viewpoints of informed individuals. Finally, in the quantitative phase, Confirmatory Factor Analysis (CFA) was applied to examine the relationships between variables coherently and present the final model. Different study phases are explained in detail as follows.

#### Phase 1: literature review

We used relevant keywords, including “responsiveness,” “health system,” “modeling,” “education,” “medicine,” and “healthcare organization,” in searching for relevant documents in databases such as Web of Science, Scopus, PubMed, Google Scholar, Iranmedex, SID, and Irandoc. We used a uniform data collection form to reduce probable bias and maintain the content’s integrity, reliability, and validity. Based on the research questions, retrieved data was tabulated to contain information such as study title, authors’ names, publication year, research type, study location, and a summary of the results. The main question for the review was to determine a comprehensive set of characteristics for responsive medical education. As a result, the extracted factors were categorized into different domains and organized in a proper format to be presented to the members of an expert panel.

#### Phase 2: Expert Panel

In this phase, researchers aimed to identify the features influencing medical education responsiveness. Therefore, extracted features from the literature review were assessed by expert panel members for transparency, applicability, and compatibility with the country’s educational system. Accordingly, some of them were omitted or revised to comply with the existing condition. Members of the expert panel were selected among individuals with sufficient knowledge and experience in planning and policy-making in different fields, including healthcare management, medical education, and curriculum development. Panel composition based on the individual’s expertise and affiliated organization is depicted in Table 1.

**Table 1:** the Characteristics of Study Participan

Characteristics		Count (n)	Distribution (%)
Gender	Male	2	16.6
	Female	10	83.4
Age	30–40	3	25
	40–50	8	66.6
	≥ 50	1	8.4
Job title	Postgraduate Studies management	2	16.8
	Management of Medical Education Research and Development	1	8.3
	Continuing Medical Education Management	1	8.3
	Secretariat of Academic Affairs	4	33.3
	Health services management	4	33.3
Length of service	< 10	2	16.8
	10–20	5	41.6
	≥ 20	5	41.6

**Fig. 1:**
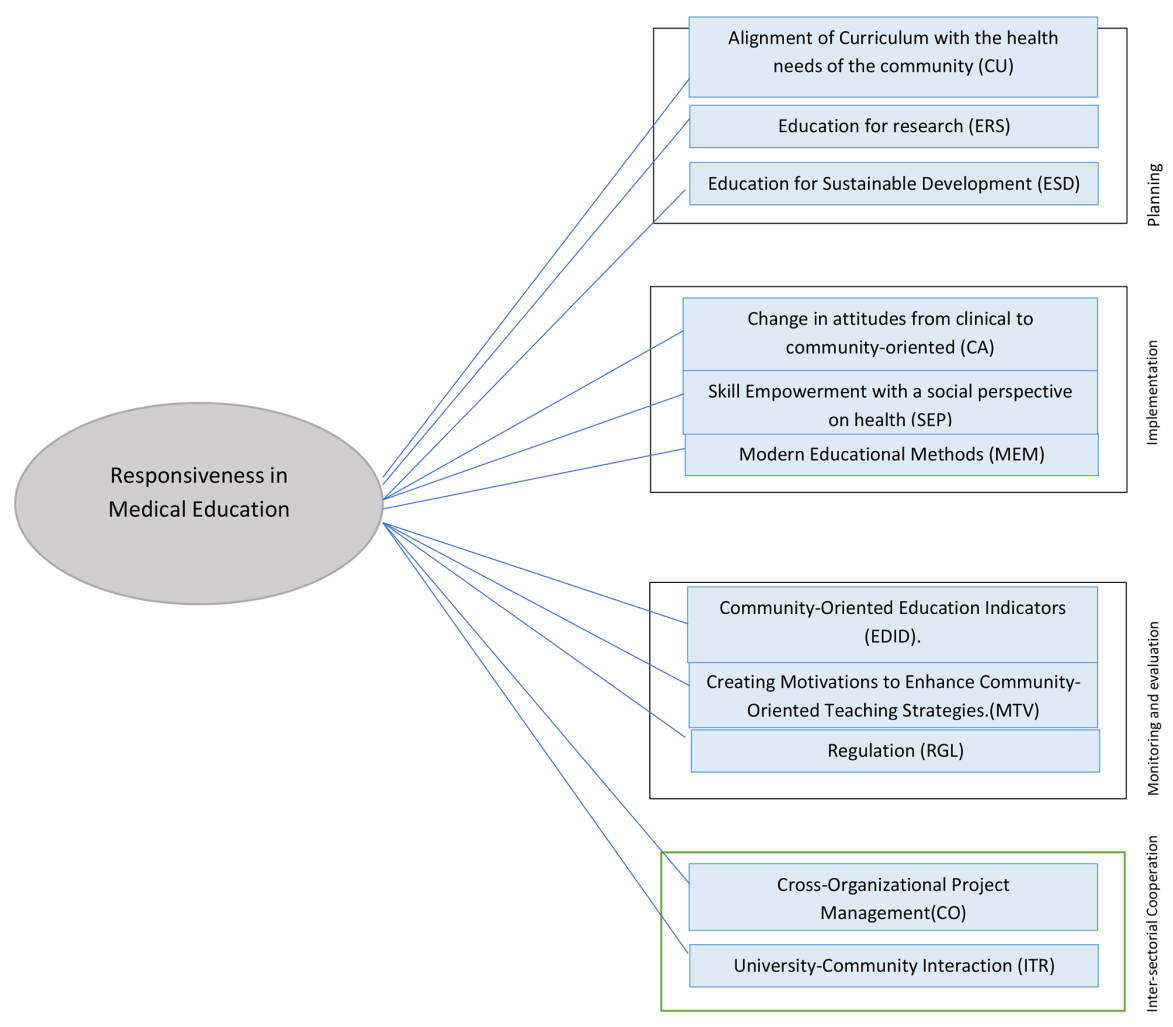
Components of responsive in healthcare in education

Before commencing the sessions, pre-session coordination was conducted, and after providing necessary explanations regarding research objectives, participants’ viewpoints were recorded only after obtaining their informed consent. The initial model formulation and relationships between study dimensions were based on the participant’s understanding of the subject and their opinions in the expert panel. Furthermore, efforts were made to include the most significant components in the conceptual model to ensure that the modeling process could yield desirable results by maintaining a manageable sample size. Ultimately, the themes and concepts of the literature review were compiled and categorized by experts. Figure 1 depicts a conceptual model of the study, resulting from a comprehensive analysis of existing literature and conceptualization by an expert panel.

Based on the literature, we suggested the following hypotheses:

H1: Alignment of curriculum with the health needs of the community has a significant impact on the responsiveness of medical education.

H2: Education of research has a significant impact on the responsiveness of medical education.

H3: Education for sustainable development has a significant impact on the responsiveness of medical education.

H4: Change in attitudes towards community-oriented approaches has a significant impact on the responsiveness of medical education.

H5: Skill Empowerment with a social perspective on health has a significant impact on the responsiveness of medical education.

H6: Modern educational methods have a significant impact on the responsiveness of medical education.

H7: Developing community-oriented education indicators has a significant impact on the responsiveness of medical education.

H8: Creating motivations to enhance community-oriented teaching strategies has a significant impact on the responsiveness of medical education.

H9: Regulation has a significant impact on the responsiveness of medical education.

H10: Cross-organizational project management has a significant impact on the responsiveness of medical education.

H11: University-community interaction has a significant impact on the responsiveness of medical education.

#### Phase 3: quantitative study

This analytical phase of the study uses collected data from previous study stages. A questionnaire was developed using a literature review and the experts’ opinion, and its face validity was assessed. CFA also employed a content validity assessment. The questionnaire consisted of two parts, including demographic information and features of a responsive education system. In the second part, 11 questions were designed in 4 dimensions: planning, implementation, monitoring and evaluation, and intra-sectoral communication. We analyzed the importance of the factors influencing the responsiveness of a medical education system with a five-point Likert scale (very low, low, medium, high, and very high). In this study phase, participants consisted of all employees working in educational deputies of the university, affiliated faculties, and hospitals with necessary knowledge and expertise in the field of education, compiling and revising educational curricula, evaluating the effectiveness of education in different faculties and hospital departments, especially those engaged in the educational accreditation program. The most common sampling method in studies that employ structural equation modeling is considering the sample size 5 to 15 times greater than the number of components (15*11 = 165). Accordingly, the final sample size of 205 was achieved after considering an attrition rate of 25% [15]. The CFA technique was employed to coherently examine the relationships between variables and present the final model. This technique consists of five stages, including the formulation of an initial model, estimation of the model involving data collection and construction of variable matrices, and ultimately, evaluation of the model fit, which entails an overall assessment of the model’s goodness-of-fit, testability, and the need for modifications. Finally, using the data from this stage, a validated research model, including its dimensions and predictive components, was presented [16].

### Data Analysis

In the quantitative phase, Confirmatory Factor Analysis (CFA) was used in software R version 3.2.4 at a significance level of 0.05. CFA is a multivariate statistical method that establishes a specific relationship among a set of seemingly unrelated variables under a hypothetical model [17].

## Results

Regarding the 32 articles from the literature review, the following features were identified (Table 2). The characteristics included equality in education, efficiency, communication, active participation in health system development, and strengthening standards and effectiveness in educational programs.

**Table 2:** Components of a Responsive Medical education System

Components	
1. Relevance and alignment of courses with health needs. 2. Greater emphasis on collaborative and team-based care in education. 3. Increased emphasis on competency-based assessment at all levels. 4. Increased emphasis on leadership development across all levels. 5. Structured support for students in all levels of medical education transition. 6. Shared mission of social responsibility for governing bodies, accreditation, and licensing. 7. Anticipating community health needs in program mission and objectives. 8. Planning and management of university activities. 9. Educational research. 10. Equity. 11. Effectiveness. 12. Communication and active participation in health system development. 13. Strengthening standards and accreditation criteria. 14. Focus on quality of service delivery in education. 15. Identification of current and future social needs and challenges. 16. Cost-effectiveness of teaching methods. 17. Justice in education. 18. Coherence, cohesion, and focus on health system conditions and requirements.	19. Intersectoral collaboration and partnerships. 20. Process-oriented approaches. 21. Governance. 22. Alignment with graduates’ job needs. 23. Faculty capacity. 24. Alignment of educational curriculum and topics with community health needs. 25. Research-informed education using empirical evidence. 26. Culture-centered education. 27. Social determinants-based education. 28. Utilization of innovative teaching methods. 29. Strengthening of practical skills in the field. 30. Assessment indicators in education. 31. Creation of motivating factors to enhance teaching strategies. 32. Legal regulations and requirements. 33. Engagement with other sectors of society. 34. Cost-effectiveness in research and planning for quality services. 35. Coordination of inter-organizational joint projects. 36. Awareness and knowledge of faculty members and other stakeholders regarding their responsibilities in education and healthcare needs.

**Fig. 2:**
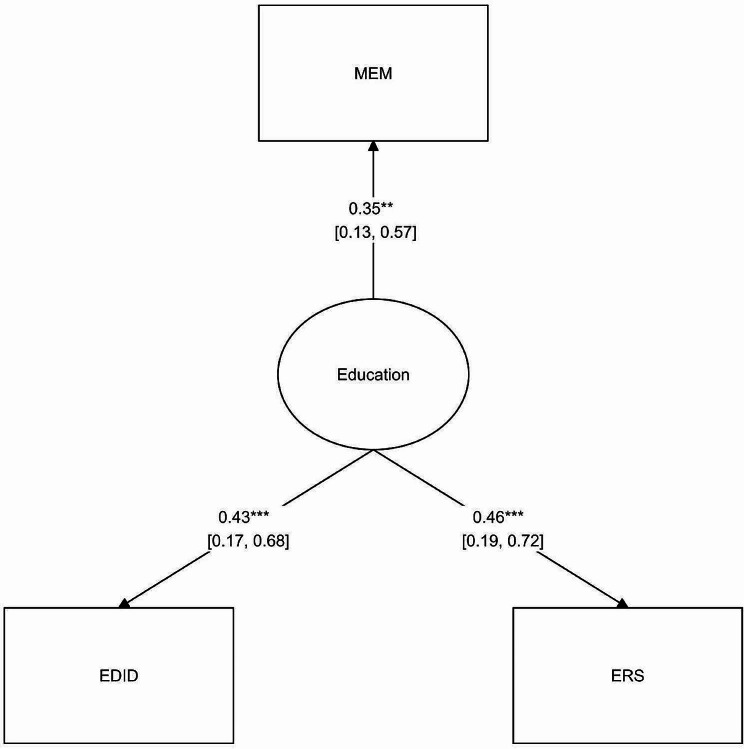
The impact of health system responsiveness components in the education domain

The fitness model was applied to examine the consistency and compatibility of the model. To do so, we evaluated the reliability and validity of the model. Likewise, the significance of the relationship between the responsiveness of an education system and its factors was investigated (Fig. 2).

Based on study findings, standard estimation coefficients of the model were at a significant level for three components: innovative educational methods, developing evaluation indicators for community-oriented education, and research-based educational planning (Table 3).

**Table 3:** The Impact of Health System Responsiveness Components in the Education Domain

Number	Component	Non-standard coefficient	Standard deviation	% confidence interval 95		Standard coefficient	*P*-value
				Upper bound	Lower bound		
1	Using innovative educational methods	1	-	-	-	0.347	-
2	Developing evaluation indicators for community-oriented education	1.958	0.93	3.78	0.136	0.425	0.035
3	Research-based educational planning	2.351	1.162	4.629	0.074	0.457	0.043

As Table 3 shows, the three mentioned components had the highest positive impact on the responsiveness of medical education. This implies that holding other conditions constant, an increase of one standard deviation in each of these components will increase system responsiveness.

Furthermore, the values calculated for indices like chi-square to degrees of freedom, GFI, NFI, and RMSEA were in the defined range, confirming that the model fitted enough (Table 4).

**Table 4:** Fitness Indices in the Model

Index	Limit	Model
χ2/df	Less than 3	2.67
RMSE	Less than 0.8	0.78
CFI	Higher than 0.90	0.89
GFI	Higher than 0.9	0.94
NFI	Higher than 0.90	0.91

## Discussion

This study aimed to identify a comprehensive set of features that significantly impact the responsiveness of the medical education system based on a mixed-method study to propose a framework for the final model. Based on the findings and reports, the practical components of responsiveness in education have been categorized into four dimensions: planning, implementation, monitoring and evaluation, and intersectoral collaboration. Among these, research-based educational planning and fulfilling an educational role, similar to therapeutic and research roles, require scientific evidence, as decisions based on personal opinions and beliefs can lead to educational deviations. Responsive education in the medical sciences represents planning based on understanding and responding to the community’s health needs and preparing students for work and service provision to the community. This finding is consistent with various studies, including those by Amir Esmaeili et al. [18] and Yaman et al. [12]. In explaining the findings, it can be said that responsive education directs all educational activities toward the training of professionals capable of meeting the health needs of the target community, and this is achieved through direct and continuous engagement of students with the community at various levels. However, since there is a gap between the current state and the desired state in the medical education system, and the clinical skills and competencies of graduates alone are not sufficient to meet the needs and expectations of the target community, universities can, by aligning with national and regional priorities, improve educational planning and enhance the quality of education and professional competencies of graduates, create an opportunity to respond to the expectations and needs of the general public, and foster greater interaction between universities and the community [19]. A study by Mullan et al. found that effective educational planning is crucial in addressing the community’s health needs. The study emphasized the importance of evidence-based educational planning to ensure that educational programs align with the specific needs and priorities of the target population [20]. A research study by Grol et al. highlighted the significance of implementation in responsive education. The study emphasized the need for effective implementation strategies to translate educational plans into action and ensure that educational interventions are delivered in a manner that effectively addresses the identified health needs [21]. Monitoring and evaluation are essential components of responsive education. A study by De Allegri et al. demonstrated that continuous monitoring and evaluation of educational programs help identify areas for improvement and ensure that the education provided is responsive to the community’s health needs [22]. Intersectoral collaboration has been recognized as a critical element of responsive education. A study by Atun et al. found that collaboration between educational institutions, healthcare providers, and other relevant sectors enhances the effectiveness of educational interventions and promotes a holistic approach to addressing health needs [23]. These studies further strengthen the argument that the four dimensions of planning, implementation, monitoring and evaluation, and intersectoral collaboration are vital components of responsive education in the medical sciences.

According to the findings, responsiveness in the medical education system, similar to other domains, has been recognized as a key objective at the global level. One tangible aspect of this responsiveness is the utilization of innovative teaching methods in this field, aiming to train competent and committed physicians who are responsive to the needs of society. This social responsiveness strives to ensure equitable access to education, quality of education, effective interactions between clinical practice and educational settings, and ultimately, maximizing the efficiency of healthcare services provision. In this regard, responsiveness in the education sector serves as a criterion for evaluating and measuring the responsiveness of educational institutions to the needs of the community. It is a social responsibility for universities and educational groups to pay attention to the needs and expectations of the community, and they should actively participate in the development of the healthcare system by adhering to fundamental principles and employing innovative teaching methods that focus on improving individuals’ rational behavior with new and novel ideas. These findings are consistent with the studies conducted by Bolen et al. [24] in a similar context. A study by Chen et al. (2017) emphasized the significance of social responsiveness in medical education. It highlighted the need for medical schools to incorporate social accountability principles to ensure that graduates are trained to meet the population’s healthcare needs and address health disparities [25]. In a research article by Dornan et al., the authors discussed the role of innovative teaching methods in medical education. The study highlighted the benefits of incorporating active learning approaches, such as problem-based learning and simulation-based training, to enhance students’ clinical reasoning skills and ability to respond effectively to real-world healthcare challenges [26]. Burch et al. conducted a study that explored the community engagement’s impact on medical education. The findings revealed that the active involvement of students in community-based projects and interactions with diverse populations contributed to their understanding of societal health issues and fostered a sense of social responsibility [15]. A systematic review by Li et al. examined the effectiveness of innovative teaching methods in medical education. The study found that incorporating technology-enhanced learning, such as virtual patient simulations and online resources, improved students’ clinical knowledge, skills, and responsiveness to patient needs [27]. These studies emphasize that responsiveness in the medical education system involves utilizing innovative teaching methods to train competent and socially responsive physicians who can effectively address the community’s healthcare needs.

Based on the findings of this research, healthcare system responsiveness in the education sector should be designed and developed in a way that pays special attention to the design and formulation of educational programs based on health needs and in line with strengthening curricula. This attention should be focused on indicators that measure the number of revised educational programs based on community needs. Responsiveness to the community’s needs is a logical, continuous, and sustainable demand resulting from the dynamic interaction between education and society. These findings are consistent with the studies conducted by Moazzami et al. [28] in a similar context. Additionally, Razavian examined various dimensions of medical faculties’ responsiveness to society and concluded that a responsive medical faculty prioritizes the revision of educational, research, and service content based on the community’s health needs and in accordance with ethical principles and standards [29]. This finding aligns with the central focus of this research. In line with our findings, a study by Frenk et al. emphasized the need for educational healthcare programs to be responsive to the changing health needs of the population. The study highlighted the importance of aligning curricula with current health challenges and ensuring that graduates are equipped with the necessary skills and knowledge to address these needs effectively [30]. In a research article by Cullen et al., the authors discussed the importance of community needs assessment in designing educational programs in healthcare. The study emphasized that understanding the community’ specific health needs and priorities is crucial for developing responsive educational curricula and ensuring that graduates are prepared to meet those needs [31]. A study by Frankish et al. explored the concept of sustainable responsiveness in healthcare. The findings highlighted the importance of ongoing assessment and adaptation of educational programs to address emerging health issues, promote innovation, and ensure the sustainability of healthcare system responsiveness [32]. In a systematic review by O’Neill et al., the authors examined approaches to developing responsive healthcare curricula. The study identified the inclusion of community needs assessments and the continuous evaluation and revision of educational programs as key elements in promoting responsiveness in healthcare education [33]. These findings support the argument that healthcare system responsiveness in the education sector should focus on designing educational programs based on health needs and strengthening curricula to ensure graduates are prepared to address the evolving health challenges of the community.

The healthcare system of the Islamic Republic of Iran is recognized, based on existing top-level documents, particularly overarching policies, as the responsible authority for the education and training of human resources in the health sector. With the considerable expansion of medical universities and higher education institutions in the country’s health sector, suitable infrastructure exists for the quantitative and qualitative improvement of higher education in health. To benefit from this infrastructure, developing a clear roadmap based on evidence and top-level documents is necessary. In this regard, the comprehensive Higher Education Plan of the healthcare system, aligned with the goals of the Healthcare Transformation Program, is a strategic document based on top-level documents, including Iran Vision 1404, the comprehensive scientific map of the country, the comprehensive scientific health map, and the healthcare system transformation program. On this basis, the Ministry of Health, Treatment, and Medical Education has presented transformation and innovation packages for medical education to be responsive. One of the key aspects highlighted is that education should be responsive and justice-oriented, ensuring that higher education programs in the health sector align with the needs of society. This will create a conducive environment for the growth and development of students with diverse scientific, cultural, and social abilities. Another essential aspect presented is the design of a system that creates appropriate sensitivity and motivation for policymakers, stakeholders, faculty members, students, and service providers to meet the real needs of the community better. These findings are consistent with the results of this study [34]. A study by Jaspers et al. emphasized the need for higher education programs in the health sector to be responsive to societal needs and promote social justice. The authors discussed the importance of aligning educational goals, content, and teaching methods with the principles of equity, diversity, and inclusion [35]. In a research article by Barry et al., the authors highlighted the significance of creating an inclusive learning environment in medical education. The study emphasized acknowledging and valuing students’ diverse backgrounds, abilities, and perspectives to promote a socially just and responsive educational experience [36]. A study by Mann et al. explored the role of policymakers in promoting responsive medical education. The findings emphasized the importance of engaging policymakers in the educational process and fostering their understanding of the community’s specific health needs and priorities to drive meaningful reforms [37]. These studies highlight that education in the health sector should be responsive, justice-oriented, and aligned with the needs of society. It highlights the importance of creating an inclusive learning environment and fostering collaboration among policymakers, stakeholders, faculty members, students, and service providers to ensure the educational system effectively addresses the community’s real needs.

## Conclusion

Establishing and developing responsiveness in the medical sciences education system requires implementing innovative educational methods that train specialized and responsive healthcare professionals. These professionals need to comprehensively understand multidimensional health concepts and effective strategies for achieving them. By doing so, the education system can take practical and responsible steps towards training competent and committed physicians who align with the needs of society. To further enhance responsiveness, it is crucial to prioritize research-based educational planning and the development of community-oriented evaluation indicators. These indicators are vital in assessing the number of revised educational programs that address societal needs. By incorporating research findings and feedback from the community, the medical sciences education system can continually improve its responsiveness and adapt to evolving healthcare requirements. Curriculum development emerges as a critical factor in ensuring the training of graduates who can provide high-quality services and meet defined standards. By focusing on the key elements in revising medical education programs, the education system can effectively address societal needs and respond to changing disease patterns and emerging health priorities. Understanding and prioritizing these elements is essential to fostering a responsive medical education system.

## Data Availability

The datasets used and/or analyzed during the current study available from the corresponding author on reasonable request. The entire dataset is in Farsi language. The Data can be available in English language for the readers and make available from the corresponding author on reasonable request.
